# Laws Regulating the Practice of Medicine in Georgia

**Published:** 1874-04

**Authors:** Thomas H. Baker

**Affiliations:** Representative from Bartow County.; Cartersville, Ga.


### A CIRCULAR.

*To the Medical Profession of Georgia:*

Having had reason to believe that the recent movement in the Legislature of Georgia, for the repeal of the laws upon the subject of the practice of medicine, has been misunderstood by a portion of the profession, and perhaps by others who have not investigated the subject, I have regarded it as advisable to make the following explanation :

Having had my attention directed to one feature of this legislation, through the efforts of the Allopathic Board, by publication in the newspapers of the State to enforce its claims to require all persons offering to practice regular medicine, with or without diplomas, to obtain a license from said board, (with fees for its benefit attached,) I found, upon investigation, that this claim was not only apparently sustained, but that the *same* board was authorized to license any individual to practice medicine, or special branches of medicine, without regard to previous training, or evidence of having had opportunity of qualification, and that even a single member of said board had the power of granting licenses in the same way, in the intervals of the sessions of the board, with power to revoke any or all of such licenses at pleasure. I found not only this board possessing powers which, if exercised, would be annoying and oppressive to the regularly educated members of the profession, and productive of great irregularities in the practice of medicine, and opposed to the spirit of the laws regulating the course of instruction in the medical colleges of this and other States, but I ascertained that this was a *part* of a system of laws which had been gradually engrafted upon the original law providing for this board, nearly fifty years since, and which established by special statute all forms of irregular practice, whether Eclectic, Botanic, Homeopathic, or other special system of quackery, either by a board or by the legal privilege of practicing, upon anything that might be called a diploma from some institution (mythical or otherwise) somewhere in the world, said to represent said system. Besides this, I also discovered that in the practical enforcement of the law as it was, it would but serve to annoy, to inconvenience, to exact money from, and to humiliate good men, whilst impostors and charlatans of every grade were only required to assert that they did not belong to this or that school, and the law could not reach them, and at the same time this law provided for the manufacture of an inferior grade of licensed doctors, without diplomas, to be carried as a dead weight by the regular profession.

Knowing that a law which required graduates in medicine to obtain a license from a board which was empowered to grant licenses equal to their own, under the law, to all persons, at their discretion, without regard to opportunities for medical education, was oppressive and offensive to those who had obtained the degree of Doctor of Medicine at large expense of time and money, and this too coupled with pecuniary exactions for the benefit alone of said board, and desiring to break up the foundations which the law had laid for all their regular forms of medicine, and having no hope that any amendment of the existing laws could be obtained, which would in any way benefit the regular profession, or protect the people from unqualified doctors, I introduced a bill in my own behalf and in the interest of the physicians of the county of Bartow, and in accordance with the almost unanimous sentiment of the medical men in the Legislature, and, as I believed, of the State, repealing the entire law regulating the practice of medicine in Georgia. This bill passed the House of Representatives by a very large majority, and would, in my opinion, have passed the Senate, but for the most urgent and persistent efforts of a *few* members of the Allopathic Board, which I shall not characterize further than to say produced an entirely erroneous impression in regard to the objects sought to be accomplished by, and the motives which influenced, those who favored an action in regard to which there was no opportunity or attempt to canvass thoroughly the physicians of the State, but from evidences which reached me from various sections and from a large number, I had reason to believe was in accordance with the sentiments of an overwhelming majority of medical men in Georgia. I desire also to call attention to the fact that practically the Allopathic Board has not been in active existence, so far as any generally successful attempt to exercise its powers upon graduates in medicine is concerned, for certainly twenty years, until within the last year, and while nominally composed of quite a number, is really constituted of a very few, (only four being required as a quorum), the large majority of those who are said to belong to the Board, having taken no interest in, and, as it is believed, not regarding themselves responsible for, its work, and, of course, have not shared in its profits, as evinced most clearly by their failure to participate in its operations.

As conclusive evidence also that the law referred to was opposed to the sentiments of the medical profession and the people of the State alike, it has, so far as graduates in medicine are concerned, remained, to all intents and purposes, a dead letter upon the statute book, and a number of acts have, at various times, passed the Legislature relieving physicians who had not complied with its requisitions; and though the influences alluded to, coupled with a relaxation of effort upon the part of the friends of the bill, resulted in a failure to repeal the entire law, yet there was no difficulty in adopting a substitute which removed the highly offensive feature requiring graduates in medicine to obtain a license from the Board.

I would farther say that, whilst I and those with whom I had an opportunity of consulting, preferred *no law*, rather than that which discriminated against regularly educated medical men, and established all forms of irregular medicine and quackery; and while I have no confidence, in the present state of government and society, in the operation of any law which attempts to prevent the people from exercising what they regard as an inalienable right to select their medical adviser, or which is designed to prevent the exercise of the healing art, upon the part of any individual who can find people disposed to employ him, the Georgia Medical Association will probably take this subject into serious consideration at its meeting in April, and if encouraged to believe that anything can be accomplished which would benefit us or the people, may endeavor by some law to obtain at least a recognition of the fact that the regular profession alone represents anything and everything that can, in any sense, be properly called medical science. Originating as it has done and appropriating and embodying in its history, literature and practice, all that has been found to be really valuable in the way of remedies for the prevention and relief of disease. While we all know, of course, that it is impracticable to obtain any law which will prevent the practice of the various forms of irregular and counterfeit medicine, it has been suggested that in response to a united sentiment of the organized profession in the State, we might obtain an entire repeal of the present law, and secure an act providing for compulsory registration, requiring every individual who offers his services to the public as a medical man, to record under oath (with penalty attached) his name, and the authority by which he offers to practice medicine or surgery, in the office of the Ordinary or Clerk of the Superior Court of the county, and refusing recognition as an "expert" in medicine or surgery in any court of law, or in connection with any legal process, to any individual who does not furnish satisfactory evidence by such record, that he has received, legitimately, the degree of Doctor of Medicine from some regular medical college. While this would not prevent the exercise of the office of physician by unqualified persons, (which is an impossibility) the profession and the people would have an opportunity of discriminating between those who offered to practice medicine, by the character of their credentials, and the regular profession of Georgia would not be placed, as now, upon the same platform with the quacks and charlatans who are legalized upon the statute book, or be compelled to bear the burden of a law which, since the action of the Legislature at its recent session, relieving graduates from the jurisdiction of the Board, still provides for the exercise of powers antagonistic to the sentiments, ethics and progress of the profession.

As it regards that portion of the present law which requires the license of *druggists* by the Board of *Doctors*, it is only necessary to state that a large proportion of the trade in drugs in the State is in, and must of necessity remain in the hands of merchants, outside of the few cities or large towns, who know nothing of and cannot possibly be made acquainted with the art of pharmacy, and can only furnish the indorsement of those who manufacture, as the evidence upon which they offer drugs to meet the demands of the people. As to those who compound drugs, my own judgment and the general opinion of medical men is, that doctors are not competent to decide upon the qualifications of pharmaceutists, and the best plan to secure skill in this department is to require recorded evidence of either a graduation in pharmacy, or of two or more years of instruction under the direction of some well qualified pharmaceutist.

And finally, I hope it will not be regarded as inappropriate (having accidentally been rendered conspicuous and somewhat responsible in this matter) for me to urge the medical men of the State not to be drawn into any controversy upon this subject. If the *few* members of the Allopathic Board, and any of their special personal friends who have taken any interest in the late legislative action, have exhibited an undue zeal in their effort to maintain their position, and to strengthen their hold upon an unwilling profession, and if those who were the subjects of the law have been earnest in the effort to relieve themselves from what they regarded as unjust, exacting, and degrading legislation, without any compensating advantages, they should bear and forbear with each other, and, in a spirit of harmony, consider the question in all its bearings, and unite in the policy which may thus be decided upon as most highly conducive to the interests of the profession and the people.

THOS. H. BAKER, M.D.,
Representative from Bartow County.

CARTERSVILLE, March 11th, 1874.

